# Testing the effectiveness of a self-efficacy based exercise intervention for inactive people with type 2 diabetes mellitus: design of a controlled clinical trial

**DOI:** 10.1186/1471-2458-12-331

**Published:** 2012-07-04

**Authors:** Marion MP van der Heijden, François Pouwer, Arnold C Romeijnders, Victor JM Pop

**Affiliations:** 1 Department of Medical Psychology and Neuropsychology, Center of Research on Psychology in Somatic diseases (CoRPS), Tilburg University, Tilburg, the Netherlands; 2 PoZoB, Coordination Centre of Practice Nurses for South East Netherlands, Veldhoven, the Netherlands

## Abstract

**Background:**

Sufficient exercise is important for people with Type 2 Diabetes Mellitus (T2DM), as it can prevent future health problems. Despite, it is estimated that only 30-40% of people with T2DM are sufficiently active. One of the psychosocial constructs that is believed to influence physical activity behaviour, is exercise self-efficacy. The goal of this study is to evaluate a patient-tailored exercise intervention for people with T2DM that takes exercise self-efficacy into account.

**Methods/Design:**

This study is conducted as a non-randomized controlled clinical trial. Patients are eligible when they are diagnosed with T2DM, exercise less than advised in the ADA guideline of 150 min/week of moderate-intensity aerobic physical activity, have an BMI >25 and are between 18 and 80 years old. Recruitment takes place at a Primary care organization of general practitioners and practice nurses in the south of the Netherlands.

Participants are allocated to three groups: An *advice intervention* -for participants with a high exercise self-efficacy score- in which participants receive a patient-tailored exercise intervention, an *intensive intervention* -for participants with a low exercise self-efficacy score- in which participants receive a patient-tailored exercise intervention accomplished by a group based intervention, and a *control group* in which participants receive regular Dutch diabetes care. The primary outcome measure of this study is physical activity. Secondary outcome measures are health status, (symptoms of) depression, exercise self-efficacy, Body Mass Index (BMI), blood pressure and glycemic control.

**Discussion:**

We aimed to design an intervention that can be implemented in Primary care, but also to design an easy accessible program. This study is innovative as it is -to our best knowledge- the first study that takes level of exercise self-efficacy of people with T2DM into account by means of giving extra support to those with the lowest exercise self-efficacy. If the program succeeds in increasing the amount of physical activity it can be implemented in regular primary care.

**Trial registration:**

Dutch Trial Register NTR2734

## Background

Diabetes mellitus is a chronic, metabolic disorder characterized by hyperglycemia as a result of a defect in insulin secretion and/or insulin resistance [1]. In 2000 the worldwide prevalence of diabetes mellitus was 171 million. This is expected to increase up to 366 million in 2030 [2]. Approximately 90% of the people with diabetes mellitus have type 2 diabetes mellitus (T2DM) [3].

T2DM often results in (severe) micro- and macro-vascular complications, such as cardiovascular problems, retinopathy, neuropathy and kidney failure [1]. Approximately 72% of the people with T2DM have at least one of these complications [4]. Besides the burden for the patient, this also imposes a burden on the health care system: people with one or more micro- and/or macro vascular T2DM complication(s) cost 70-350% as much a year compared to patients without T2DM complications [4]. In addition, global mortality attributable to diabetes was estimated to be 2.9 million people in 2000, 5.2% of all deaths [5].

Physical inactivity is one of the major risk factors for T2DM and related complications, independent of body mass index (BMI) [6-8]. Increasing physical activity reduces the risk for T2DM and its complications by enhancing metabolic control. Moreover, appropriate levels of physical activity are associated with increased cardio-respiratory fitness, increased health related quality of life and a reduced risk of total- and cardiovascular mortality [9-11]. The American Diabetes Association advises people with T2DM to have at least 150 min/week of moderate-intensity aerobic physical activity (50–70% of maximum heart rate) to maximize the effects of physical activity. In addition, resistance training should be performed three times per week in the absence of contraindications [12].

Because of its beneficial effects, achieving and/or maintaining an appropriate level of exercise is an important goal in diabetes management [12]. Despite, it is estimated that only 30-40% of people with T2DM are sufficiently active [13,14]*.* This percentage appeared to be stable in recent decades [15] and is significantly lower compared to people without T2DM [13,16]. Physical activity levels might be increased by means of an exercise intervention. Several intervention elements are thought to increase the success rate of such an intervention. To start with, an exercise intervention should imply more than just education [17]. In addition, a patient-tailored exercise plan optimises the effect of the intervention and can increase adherence to the exercise routine [18,19]. A gradual increase of physical activity [19] can avoid injuries. Also, the use of multiple behavioural components such as goal-setting, problem solving and feedback can further increase the (long-term) success of the intervention [20]. In addition, the American College of Sports Medicine and the American Diabetes Association advised in their 2010 joint statement on Exercise & Diabetes to focus exercise interventions on self-efficacy [10].

Self-efficacy is derived from the Social Cognitive Theory, which states that a behavioural change is made possible by a personal sense of control. Self-efficacy is one of the main constructs of this theory and is the “belief in one’s capabilities to organize and execute the courses of action required to produce given attainments” [21]. Self-efficacy for exercise/physical activity (exercise self-efficacy) is believed to influence physical activity behaviour [22-24]. In people with T2DM a high level of exercise self-efficacy is thought to be predictive of exercise initiation and maintenance over time [25], and is thought to mediate the relationship between an exercise intervention and physical activity [26]. Also, an increased level of exercise self-efficacy makes it more likely that participants indeed use strategies to improve their physical activity levels [27].

A patient-tailored exercise intervention with a gradual increase of physical activity and multiple components such as goals setting, has the potential to increase the physical activity level of people with T2DM. This can be provided by means of several individual physiotherapy consultations in which a patient tailored exercise plan is provided and progression evaluated. Because of its important predictive and mediating role, exercise self-efficacy has the potential to discriminate between subjects who can be helped with such individual physiotherapy consultations, and those who are in need of extra support because of their low exercise self-efficacy. Extra support can be given by providing an additional group-based program. A group-based program gives room to verbal persuasion by the physiotherapist and/or group members and vicarious experiences which are sources of self-efficacy [28] and can therefore increase the level of exercise self-efficacy.

### Objectives and hypotheses

The goal of this study is to evaluate a patient-tailored exercise program for people with T2DM that takes levels of exercise self-efficacy into account. If the program succeeds in increasing the amount of physical activity it can be implemented in regular Primary diabetes care.

The primary outcome measure of this study is physical activity. We hypothesize that the intervention will significantly increase the level of physical activity in the intervention groups compared to the control group. In addition, we will examine which determinants contribute to a successful change of the amount of physical activity. Most literature seems to agree that a high age [15,16,22,29,30], low education [15,22,30] and being female [16,22,30], are associated with a lower level of physical activity. Although a higher BMI seems to correlate with physical activity among adults [22], the results of studies on people with T2DM are conflicting [13,15,29]. In addition a low exercise self-efficacy [22], low social support [22], a depressed mood [31] and a type D personality [32] are thought be determinants of a low level of physical activity. We therefore hypothesize that a young age, high education, being male, low BMI, a high level of exercise self-efficacy, a high level social support, the absence of a depressed mood and not having a type D personality contribute to a successful change of the level of physical activity.

Secondary outcome measures are health status, (symptoms of) depression, exercise self-efficacy, BMI, blood pressure and glycemic control.

In line with the Look AHEAD trial [33] and the DARE study [34] we hypothesize that our intervention will significantly improve the physical component of health status - but not the mental component-, compared to the control group.

In two systematic reviews [35,36] on the effects of physical activity on depressive symptoms in elderly people, it was found that increased levels of exercise can lower depression rates and reduce depressive symptoms in the short term. In accordance, we therefore hypothesize that the intervention of the current study will significantly reduce depressive symptoms, compared to the control group.

The intervention is thought to provide participants personal mastery experiences which is a source of self-efficacy [28]. We therefore hypothesize that the intervention will significantly increase exercise self-efficacy, compared to the control group. We additionally hypothesize that the change of exercise self-efficacy will be larger in those with low exercise self-efficacy at baseline as they will receive additional support.

Glycemic control is assessed by means of haemoglobin A_1C_. As physical activity is thought to improve glycemic control [37-39] we hypothesize that our intervention will significantly reduce haemoglobin A_1C_, compared to the control group.

Based on literature, an effect of increased physical activity on body mass or BMI can not be expected [37,38]. We therefore hypothesize that the intervention will not lead to significant decrease in BMI, compared to the control group.

Literature on the effect of physical activity on blood pressure is somewhat ambiguous. In the review of Thomas *et al.*[37] no effect on diastolic of systolic blood pressure was found. The American College of Sports Medicine and American Diabetes Association concluded in their joint statement on Exercise and Diabetes that only slight reductions of systolic blood pressure can be expected [10]. However, in a meta-analysis by Snowling *et al.*[40] small to moderate effects of aerobic or a combination of aerobic and resistance exercise on blood pressure were found. We therefore hypothesize that the intervention of the current study will significantly reduce systolic blood pressure in those with an elevated blood pressure, compared to the control group.

Finally, diabetes self-efficacy and quality of sleep are explorative outcome measures. We therefore have no hypotheses regarding these outcome measures.

## Methods/Design

### Study design

This study is conducted as a non-randomized controlled clinical trial. Participants are allocated to three groups: An *advice intervention* in which participants receive a patient-tailored exercise intervention, an *intensive intervention* in which participants receive a patient-tailored exercise intervention accomplished by a group based intervention, and a *control group* in which participants receive regular Dutch diabetes care.

### Eligibility

Patients are eligible when they are diagnosed with T2DM, exercise less than advised in the ADA guideline of 150 min/week of moderate-intensity aerobic physical activity [12], have an BMI >25 and are between 18 and 80 years old.

Patients are excluded when they recently participated in an exercise program on an indication other than diabetes or suffer from a serious diabetes complication or other disabling co-morbidity (e.g. unstable angina pectoris, heart failure, extremely high blood pressure (systolic > 200, diastolic > 120), cerebrovascular accident, serious neuropathy, kidney failure, diabetic foot ulcer(s), proliferative retinopathy, a serious form of cancer, orthopaedic constraints, serious exertion hypertension, unstable coronary ischemia). Patients with silent myocardal ischemia are also excluded. Silent myocardial ischemia is defined as an objective documentation of myocardial ischemia in the absence of angina or angina equivalents [41]. The prevalence of silent myocardial ischemia varies from 9% up to 57% in people with T2DM [42], and can be verified by a electrocardiogram exercise stress test.

### Recruitment

Participants are recruited in rural areas surrounding the city of Eindhoven, the Netherlands. Recruitment takes place at a primary care organization of general practitioners and practice nurses in the south of the Netherlands, PoZoB (Praktijkondersteuning Zuid-oost Brabant). Eligibility is checked by the general practitioner and/or the practice nurse.

### Allocation procedure

For practical reasons, seven areas are pointed out as ‘intervention area’. People outside these areas are invited for the control group.

Participants of the intervention are allocated to the intensive intervention or the advice intervention after filling out the baseline questionnaire. Allocation is based on their score on the Exercise Self-efficacy Scale (ESS) that has been developed by Bandura [43]. Since there is to the best of our knowledge no other study that uses the ESS to discriminate between people with T2DM in a exercise intervention, we use the median score on the ESS to split the group of intervention participants approximately in two equal halves: Participants with a score between 0 and 40 are allocated in the intensive intervention. Participants with a score between 41 and 100 are allocated to the advice intervention.

### Intervention

The interventions’ duration is 36 weeks, with a follow-up period of one year.

In the advice intervention (for participants with a high exercise self-efficacy), the intervention starts with an intake consultation with a physiotherapist. During this intake, information about the intervention and ‘Diabetes and exercise’ is provided. Next, the current physical condition in terms of aerobic power, muscle strength, blood pressure, BMI and waist-circumference is established. Exercise tolerance is assessed with the six minute walk test [44]. The test is suitable for elderly [45] and a proper reflection of activities of daily living [46]. Muscle strength is assessed with one repetition maximum tests for the abdominal, back, arm and leg muscles. One repetition maximum tests are a widely spread and valid way to asses muscle strength in vivo [47]. In addition, the goals and the exercise preferences of the participant are discussed. Subsequently, a patient-tailored exercise plan is drafted. The exercise plan consists of a combination of aerobic and resistance exercise, replenished by balance and flexibility drills, with the aim of exercising three hours a week, with a gradual increase of intensity, difficulty and duration. When applicable, at-home drills are taught. An information folder with the exercise plan, written prescription of the drills, an exercise journal and information on ‘diabetes and exercise’ are provided. Also, an information session with a dietician is scheduled, in order to provide information on nutrition, diabetes and exercise.

In consultations after 4, 12, 24 and 36 weeks, progression is assessed and the exercise plan is evaluated. Also, feedback is given and possible barriers to exercise and their conceivable solutions are discussed. If necessary, the exercise plan can be adapted. In the consultations after 24 and 36 weeks, additionally, relapse prevention and the continuation of exercise after the intervention are discussed.

Participants of the intensive intervention (for participants with a low exercise self-efficacy) receive a similar intervention as do participants of the advice intervention, but in addition they receive physiotherapist guided group training. Groups consist of eighth participants maximally. The 24 group trainings contain aerobic, resistance, balance, and flexibility drills. From week one up to week eight there are two group sessions of one hour a week. From week nine up to week sixteen there is one group session a week. During these sixteen weeks, the participant is expected to exercise home-based to meet up the requirements of their exercise plan. After, participants are expected to follow up their exercise plan home-based.

### Data collection

The assessments of the self-reported data take place at baseline, at 36 weeks, at 1 year and at 1 year and 36 weeks. The intervention participants receive two additional questionnaires at 12 weeks, and 24 weeks. Questionnaires will be sent out by post, along with a stamped ballot paper envelope.

Biomedical parameters BMI, blood pressure and haemoglobin A_1C_ are assessed as part of regular care up to four times a year. In addition, intervention participants will receive a BMI and blood pressure assessment at baseline, at 12 weeks, at 24 weeks, and at 36 weeks during their physiotherapy consultations (Table 1).

**Table 1:** Measurements and time points

	Intervention group						Control group					
	T0	T1	T2	T3	T4	T5	T0	T1	T2	T3	T4	T5
**Concept (questionnaire)**												
Demographic variables	x						x					
Physical activity (SQUASH)	x	x	x	x	x	x	x			x	x	x
Health Status (SF12)	x	x	x	x	x	x	x			x	x	x
Depressive symptoms (PHQ9)	x	x	x	x	x	x	x			x	x	x
Exercise self-efficacy (ESS)	x	x	x	x	x	x	x			x	x	x
Social support	x						x					
Type D (DS14)	x						x					
Diabetes self-efficacy	x	x	x	x	x	x	x			x	x	x
Quality of sleep	x	x	x	x	x	x	x			x	x	x
Patient satisfaction		x	x	x								
**Clinical measurements**												
BMI	x	x	x	x			Part of regular diabetes care*					
Bloodpressure	x	x	x	x			Part of regular diabetes care*					
Glycemic control (hemoglobin A_1C_)	Part of regular diabetes care^*^						Part of regular diabetes care*					

To - baseline; T1 - after 12 weeks; T2 - after 24 weeks; T3 - after 36 weeks (end of intervention); T4 - after 1 year (post intervention); T5 - after 1 year and 36 weeks (post intervention); SQUASH - Short QUestionnaire to ASsess Health enhancing physical activity; SF12 - Short Form Health Survey 12; PHQ9 - Patient Health Questionnaire 9; ESS - Exercise self-efficacy scale; DS14 - Type D Scale-14; * up to 4 times a year.

### Outcome parameters

#### Primary outcome measure

##### Physical activity

The level of physical activity is assessed by means of self-report using the Short QUestionnaire to ASsess Health enhancing physical activity (SQUASH). This questionnaire has been proved to be a reliable and valid measurement in adults [48]. Participants are asked to indicate their level of physical activity during an average week in the past month, with regard to activity at work (if applicable), commuting activities (if applicable), household activities (if applicable), and leisure time. The frequency of activities (days/week), as well as the duration (average time/day) and experienced intensity of these activities (light, moderate, vigorous), are measured. Each activity is represented by a metabolic equivalent (MET). MET expresses the cost of energy from physical activities as multiples of the resting metabolic rate. Activities can be divided into three categories of intensity based on their MET: light (2 to <4.0 METs), moderate (4.0 to <6.5 METs) and vigorous (≥ 6.5 METs) intensity [48].

#### Secondary outcome measures

##### Health status

Health status is assessed using the Short Form Health Survey 12 (SF12), a shortened version of the Short Form Health Survey 36 [49]. The SF12 is a self-reported questionnaire and consists of twelve items with two component summary scores: physical health (PCS) and mental health (MCS). Both components are scored on a scale from 0 to 100, with a higher score representing a better health status [50].

##### Depressive symptoms

Depressive symptoms are assessed with the self-reported Patient Health Questionnaire 9 (PHQ9). The questionnaire contains nine items derived from the Diagnostic and Statistical Manual of Mental Disorders, Fourth Edition (DSM-IV), related to the past two weeks. The items are scored on a four point scale, ranging from 0 (not at all) to 3 (almost every day) [51].

##### Exercise self-efficacy

Exercise self-efficacy is assessed with the ESS [43]. This self-reported questionnaire contains eighteen items that describe situations during which it could be difficult to adhere to an exercise routine, for example: “when I am feeling tired” or “during bad weather”. Participants are asked to rate their degree of confidence to continue with regular exercise in the listed situations. The ESS uses a 100-point scale for each item, ranging from 0 (“I cannot do this at all”) to 100 (“I am certain that I can do it”), the higher scores reflecting higher levels of exercise self-efficacy. The total ESS score is calculated as the mean of all the items. The ESS appeared to have a single factor solution [52,53]. The questionnaire is positively correlated with the frequency of exercise [52], has no floor or ceiling effects, good internal consistency with an Cronbachs α coefficient of 0,95 and has good responsiveness to change [53].

Since a relatively high percentage of people with T2DM are retired, the original item “When I am feeling under pressure from work” is in the current study adapted to “When I am feeling under pressure”.

##### BMI, blood pressure and hemoglobin A_1C_

Biomedical parameters (laboratory tests and physical examination) are assessed as part of regular care up to four times a year and performed by the Diagnostic Centre in (areas surrounding the city of) Eindhoven, the Netherlands. To assess glycemic control we retrieve data from their files on haemoglobin A_1C_. In addition, data on BMI and blood pressure (systolic and diastolic) are retrieved from the files. Participants of the intervention receive additional BMI and blood pressure measurements as part of the physiotherapist consultations.

#### Explorative outcome measures

##### Diabetes self-efficacy

Twelve self-reported items are used to quantify diabetes self-efficacy. The participants are asked to rate their level of confidence to execute diabetes self-care behaviours. The items are scored on a six-point likert scale, from 0 (‘totally disagree’) to 5 (‘totally agree’) and concern ‘prevention of high blood sugars’, ‘healthy diet’, ‘avoid/reduce obesity’, ‘proper use of medication’, ‘enough exercise’, ‘no smoking’, ‘no alcohol’, ‘ask for help when necessary’, ‘follow up appointments with caregivers’, ‘follow up life-style advice in special situations’, ‘ask for clarification regarding T2DM when things are unclear’ and ‘accepting T2DM’.

##### Quality of sleep

Thirteen self-reported items assess various aspects of quality of sleep, related to the past week. The items concern the length of sleep, difficulty of getting to sleep or staying a sleep, overall quality of sleep, resting during the day, sleeping during the day, difficulty staying awake during the day and sleep-related medications.

#### Additional measures

##### Demographic and clinical variables

Age, gender, nationality, marital status, living arrangements, education, work situation, smoking behaviour, alcohol behaviour and year of diabetes diagnosis are assessed by means of a self-reported questionnaire at baseline. Co-morbidities, medication and diabetes treatment (diet and/or tablets and/or insulin) are retrieved from (electronic) medical records when possible.

##### Social support

Social support not only has a main effect on the level of physical activity, a study with older adults with multiple illnesses showed that social support also has a synergistic effect with exercise self-efficacy [54]. Social support is measured by four questions that are rated on a five point scale (0 = no support, 4 = much support).

##### Type D personality

People with a Type D personality have a general tendency towards increased negative affectivity and inhibit these emotions in social situations. People with a type D personality adhere less to the physical activity norm [32].

Type D personality is measured by the Type D Scale-14 (DS14). The DS14 comprises fourteen items and is scored on a five point scale (0 = false, 4 = correct). The DS14 consists of two subscales for which sum scores are calculated: negative affectivity and social inhibition. Both scales are internally consistent (Cronbachs α coefficient of 0.88 and 0.86 respectively) and are independent of mood and health [55].

##### Patient satisfaction

Participants of the intervention are asked to rate their satisfaction with treatment by means of a self-report questionnaire regarding the organization of the intervention, supervision of the physiotherapist and the content of the intervention. In addition, it is assessed whether the participant intends to continue the level of physical activity after the intervention.

### Statistical analyses

#### Sample size and power calculation

The intervention of this study consists of two groups (an intensive group and an advice group) to which participants are allocated based on their exercise self-efficacy score. Therefore, for analyses, the control group participants will also be allocated to a low exercise self-efficacy control group (ESS ≤40) and a high exercise self-efficacy control group (ESS >40).

Fisher’s exact test was used to calculate the number of subjects that have to be included in this study. We expect 50% of the intervention participants and 25% of the control group participants to meet up with the requirement of 150 minutes a week of moderate intensity physical activity at the end of the intervention (36 weeks). Taken into account a power of 0.9, an alpha of 0.05, an expected drop-out rate of 25% in the intervention and 50% in the control group, 94 participants need to be included in the intensive intervention group, 94 participants in the advice intervention group, 142 participants in the high exercise self-efficacy control group, and 142 participants in the low exercise self-efficacy control group. To be able to perform cross-validation and perform analysis in (equally sized) subgroups, an inclusion of 366 participants in the intervention and 586 participants in the control group is desirable.

#### Planned analyses

Analyses will be performed using the latest version of the Social Package for Social Sciences (IBM, Chicago, Illinois, USA). Assumptions for parametric analyses will be checked for all analysis, and tests will be performed with a significance level of 5%. Differences in patient characteristics between groups will be checked be means of T-tests and X^2^ tests, or there non-parametric equivalents.

Repeated measures analysis of variance and multilevel analysis for longitudinal data will be used to examine longitudinal differences between groups on primary and secondary outcome measures. To test which determinants contribute to a successful change in the level of physical activity, linear/logistic regression analyses will be used. Participants will be analysed within the group to which they were allocated.

### Ethical principles

The study protocol has been approved by the medical ethical committee of the St. Elisabeth Hospital, Tilburg, the Netherlands. Written consent will be obtained from all participants.

## Discussion

The number of people with T2DM is rapidly rising up to 366 million in 2030 [56]. As physical activity can - among other things- improve glycemic control, people with T2DM are advised to have at least 150 min/week of moderate-intensity aerobic physical activity [12]. Unfortunately only 30-40% of people with T2DM are sufficiently active [13,14]. An important predictor of exercise initiation and maintenance in people with T2DM, is exercise self-efficacy [25]. We therefore designed a patient-tailored intervention that aims to increase the level of physical activity of the participants, in which extra support is given to those with the lowest exercise self-efficacy.

In this study we aim not only to design an intervention that can directly be implemented in primary care, but also to design an easy accessible program. We chose to use a multi-centre approach with seven physiotherapist practices, so that participants’ travelling time is minimized. This approach also allows participants to meet other participants from the neighbourhood, which might stimulate the continuation of exercise after the intervention. Also, the physical condition and preferences of the participants are taken into account in the patient-tailored exercise plan. As a result the exercise plan is suitable for the participant, the risk of injuries is minimized, and adherence might be increased.

This study has several strengths. First, this study is innovative as it is -to the best of our knowledge- the first study that takes levels of exercise self-efficacy of people with T2DM into account, giving extra support to those with the lowest exercise self-efficacy. Secondly, the large number of participants that will be included. As the intervention is embedded in regular diabetes care, we are confident that we will be able to include the required number of participants. Thirdly, as a large portion of the studies on physical activity in people with T2DM focuses on clinical outcomes, we also included psychosocial outcomes such as perceived health status and depressive symptoms. Finally, this study focuses on primary care patients. Primary care patients tend to have less severe co-morbidities compared to secondary care patients, so prevention of future co-morbidities might be possible.

Several limitations of the design of this study also need to be addressed. This study is not randomised. This may lower the validity of our findings. However, by using a matched control group (age and gender) and correcting for baseline differences, we will try to minimize the shortcoming of a non-randomised study. Another limitation is the determination of the ESS cut-off point for participant allocation, as it is based on a median score. It is possible that post hoc analysis might suggest that other cut-off points should have been used. Also, glycemic control will be measured only as part of regular diabetes care, as are BMI and blood pressure for the control group participants. As a consequence, the measurement time points of the questionnaires may not correspond with these laboratory assessments. However, BMI and haemoglobin A_1C_ are rather stable biological markers of T2DM and we aimed to design an easy accessible intervention, which includes minimization of the participants’ burden. Finally, for practical reasons the measurement of physical activity is by means of self-report. Self-reported physical activity tends to result in an overestimation of levels of activity and different activity patterns compared to more objective estimates [57]. Yet, the SQUASH shows moderate correlations with data from accelerometer measurements [48].

## Conclusion

In conclusion, the need to increase the level of physical activity in people with T2DM is evident, in which exercise self-efficacy can play an important role. In the present study we will evaluate a patient-tailored exercise program for people with T2DM that takes levels of exercise self-efficacy into account.

## Competing interests

The authors declare that they have no competing interests.

## Authors’ contributions

All authors contributed to the design of the study. VP is the principal investigator of the study. MH drafted the manuscript. FP, AR and VP reviewed/edited the manuscript. All authors approved the various versions including the final version of the manuscript.

## Pre-publication history

The pre-publication history for this paper can be accessed here:

http://www.biomedcentral.com/1471-2458/12/331/prepub
